# Health and Pleasure in Consumers' Dietary Food Choices: Individual Differences in the Brain's Value System

**DOI:** 10.1371/journal.pone.0156333

**Published:** 2016-07-18

**Authors:** Olivia Petit, Dwight Merunka, Jean-Luc Anton, Bruno Nazarian, Charles Spence, Adrian David Cheok, Denis Raccah, Olivier Oullier

**Affiliations:** 1 Imagineering Institute, Iskandar-Puteri, Malaysia; 2 City University London, London, United Kingdom; 3 Aix-Marseille University, CERGAM EA 4225, Aix-Marseille Graduate School of management – IAE, Aix en Provence, France; 4 Aix-Marseille University & CNRS, LPC, UMR 7290, FED 3C, Behavior, Brain & Cognition Institute, FR 3512, Marseille, France; 5 Kedge Business School, Marseille, France; 6 Aix Marseille University, CNRS, Centre IRMf, INT UMR 7289, Marseille, France; 7 Crossmodal Research Laboratory, University of Oxford, Oxford, United Kingdom; 8 Department of Diabetology, University Hospital Sainte-Marguerite, Marseille, France; Banner Alzheimer's Institute, UNITED STATES

## Abstract

Taking into account how people value the healthiness and tastiness of food at both the behavioral and brain levels may help to better understand and address overweight and obesity-related issues. Here, we investigate whether brain activity in those areas involved in self-control may increase significantly when individuals with a high body-mass index (BMI) focus their attention on the taste rather than on the health benefits related to healthy food choices. Under such conditions, BMI is positively correlated with both the neural responses to healthy food choices in those brain areas associated with gustation (insula), reward value (orbitofrontal cortex), and self-control (inferior frontal gyrus), and with the percent of healthy food choices. By contrast, when attention is directed towards health benefits, BMI is negatively correlated with neural activity in gustatory and reward-related brain areas (insula, inferior frontal operculum). Taken together, these findings suggest that those individuals with a high BMI do not necessarily have reduced capacities for self-control but that they may be facilitated by external cues that direct their attention toward the tastiness of healthy food. Thus, promoting the taste of healthy food in communication campaigns and/or food packaging may lead to more successful self-control and healthy food behaviors for consumers with a higher BMI, an issue which needs to be further researched.

## Introduction

Value plays an important role in motivating action, especially in regulating one’s behaviors [1]. In the context of food consumption, exercising self-control during food choices requires individuals to value healthy food positively [2]. To enhance such a valuation, recommendations for fighting obesity tend to use a “food = nutrients = health” strategy [3]. However, as Block et al. [3] correctly highlight the fact that: “*No one sits down to eat a plate of nutrients*. *Rather*, *when we sit down for a meal*, *we are seeking physical as well as emotional and psychological nourishment*.” People choose tasty food in an environment in which visual exposure to desirable (and often unhealthy) food has increased dramatically and may very well be exacerbating their food craving [4]. This could turn into a major health problem given that individuals, especially those with a high Body Mass Index (BMI) intuitively believe that healthiness and tastiness are negatively correlated [5–6]. Improving the perceived value of healthy food as a means of facilitating self-regulation may therefore be an important step when it comes to fighting overweight and obesity [7].

The unhealthy = tasty intuition (UTI) leads many people to make unhealthy food choices, and to exhibit less interest in health-related information, which therefore affects their BMI [5]. While increasing health consciousness (i.e., the degree to which people are interested in their health and motivated to engage in preventative behaviors and healthcare strategies) has a positive effect on one’s interest in health-related information, it does not counter the spontaneous and implicit UTI, nor does it affect BMI. By seeking to make healthy food choices, people may be too focused on nutrition to be able to enjoy the consumption experience itself [8–9]. For instance, dieters generally consider eating healthy food as a constraint and may fail to control-themselves fighting their own urges to consume unhealthy foods [10–12]. These difficulties in self-control can also be explained by the fact that, on average, at the brain level, tastiness is processed earlier than healthiness during food choice [13]. For this reason, healthiness may have less weight than tastiness when people choose between unhealthy and healthy options [13].

The relation between self-control and the value of healthiness has been extensively documented at the neural level [2, 14]. For instance, Hare et al. [2] demonstrated that activity in a reward-related brain area (the ventromedial prefrontal cortex, vmPFC) of so-called ‘non-self-controllers’ (i.e., those who choose liked-unhealthy food or decline disliked-healthy food) is only correlated with the taste ratings given to food items. On the other hand, activity in this brain network is also correlated with health ratings for self-controllers (i.e. those who decline liked-unhealthy items and/or choose disliked-healthy ones). These individuals also exhibit greater activation of the dorsolateral prefrontal cortex (DLPFC), an area that is involved in the presence of conflicting desires requiring self-control. It appears as though obese individuals—as opposed to those who are non-obese—also have reduced activation in this network of the brain (i.e. the left DLPFC) as well as in the orbitofrontal cortex (OFC), another reward-related brain area, whenever they inhibit urges to eat the food [15]. They also exhibit less activation in the left DLPFC compared to non-obese individuals when viewing food pictures [16]. Similarly, Wagner et al. [14] report that when depleted dieters (i.e., those who have completed a task that is known to result in self-regulatory depletion; see [17] for a reconsideration of ego-depletion) were compared to non-depleted dieters, greater activity was observed in the orbitofrontal cortex (OFC) in response to viewing desirable food pictures. Moreover, a decrease of brain’s functional connectivity was documented between this area and an area involved in self-control (the inferior frontal gyrus, IFG). Taken together, these findings suggest that people making unhealthy food choices only value the taste of food and use less self-control when making food choices. For this very reason, these individuals are more likely to succumb to temptation while on a diet [12].

However, it is possible to facilitate self-control by having people consider the healthiness of the food during food choices [18]. Such a focus of attention modulates activity in reward (vmPFC) and self-control (DLPFC, IFG) areas, leading to an increase in healthy food choices. These results therefore suggest that drawing attention to healthy food may lead to a modulation of activity in those brain areas that are involved in exercising self-control. However, for those individuals who only value tastiness, especially those with a high BMI, drawing attention to health benefits will not help them improve self-control.

Here, our objective is to assess whether drawing attention to the tastiness of healthy food modulates activity in brain areas that are used to exercise self-control and whether it increases healthy food choices in individuals with a high BMI. We systematically test: (1) Whether, during healthy food choice, activity in networks involved in self-control (i.e., DLPFC, IFG) [2, 14–16, 18–22] and areas participating in coding the gustatory and reward value of food (insula, OFC/vmPFC) [2, 14–15, 18, 20–31] increases when attention is focused on healthy food as compared to a control condition, regardless whether their attention is focused on the healthiness or the tastiness of the food; (2) Whether the BMI of participants is negatively correlated with the activity in these areas when attention is directed toward health benefits; and (3) Whether the BMI is positively correlated with activity in these areas when attention is focused on the tastiness of healthy food.

By demonstrating that the modulation in the brain’s network involved in self-control is positively correlated with BMI when attention is focused on the tastiness of healthy food, we provide evidence that high BMI individuals do not necessary have less of a capacity for activating the related brain areas. They may need to value the tastiness of healthy food in order to control their behavior. Thus, adapting healthy food campaigns to targeted audiences may constitute an effective means of fighting in some small way obesity. This hypothesis will be partially addressed by our experiment but will, of course, need further research at the multi-stakeholder level (academia, public health sector and food industry) to improve the health and well-being of overweight and obese people.

## Materials and Methods

### Ethics statement

All of the participants went through a specific medical exam and gave their written informed consent prior to taking part in the fMRI study, which received the approval of local (Aix-Marseille University ethics committee), regional (“Comité de Protection des Personnes Sud Méditerranée”), and national ethics and regulatory agencies in France (“Agence Nationale de Sécurité du Médicament et des Produits de Santé”).

### Participants

23 right-handed non-restrained individuals with normal or corrected to normal vision (10 females, mean age = 25.91, *SD =* 3.85; mean BMI = 23.47, *SD =* 2.8) volunteered to take part in the study. Participants were only eligible if they reported frequent consumption of the type of food used in the study and had no history of psychiatric or neurological conditions. Subjects received €50 in return for taking part in the study. One participant was excluded for excessive movement during functional brain scanning, which left 22 participants for final analysis.

### Stimuli

Participants rated and made decisions concerning 128 different tasty food items, including 50% junk food (e.g., chips, pizza, candy bar) and 50% healthy snacks (e.g., apple, orange, broccoli), presented as 72 dpi color pictures. Half of the food items were branded and half unbranded. The stimuli were selected from 500 food pictures that had been rated in terms of their healthiness and tastiness, by 236 participants on 7-point Likert scales, from 1 (= not at all) to 7 (= very much so) [32]. We classified the food items as healthy (vs. unhealthy), and tasty (vs. not tasty), when the mean score was significantly above (vs. below) the scale midpoint (i.e., 4). We selected a final set of 64 healthy food pictures to be used in the experiment (*M*_healthiness_ = 4.74, *SD =* .70, *Z* = 16.24, *p* < .001) and 64 unhealthy food pictures (*M*_healthiness_ = 2.64, *SD =* .46, *Z* = -45.41, *p* < .001), both categories being rated as tasty (*M*_healthy_food_ = = 4.38, *SD* = .76, *Z* = 7.68, *p* < .001; *M*_unhealthy_food_ = 4.52, *SD* = .65, *Z* = 12.29, *p* < .001).

### Task

The participants were instructed not to eat in the 4 hours prior to the experiment [2,23]. During each trial, they were shown a picture of one of the 64 selected food items and were given up to 3 s to indicate whether they would be willing to eat that item at the end of the experiment or not. The picture of the food product disappeared as soon as a response was made. Participants indicated their response on a 4-point scale: Strong No, No, Yes, and Strong Yes. Their response was displayed for 0.5 s on the screen before the next image was presented. At the end of the experiment, one of the trials was randomly selected. If the participant had declared a “Yes” or a “Strong Yes” for that item, they would get to eat the food item, while if they had declared a “No” or a “Strong No”, then they would not, and another food item was proposed instead. In either case, the participants were required to stay in the lab for 30 min after the experiment in order to eat. Note that because the participants did not know which trial would be selected, their optimal strategy was to treat each decision as if it were the only one. Thus, participants had no obvious incentive to select “Yes” or “Strong Yes” for an item that they did not like, regardless the condition. Trials were separated by a variable inter-trial interval (ITI) ranging from 5 to 9 s.

**Fig 1:**
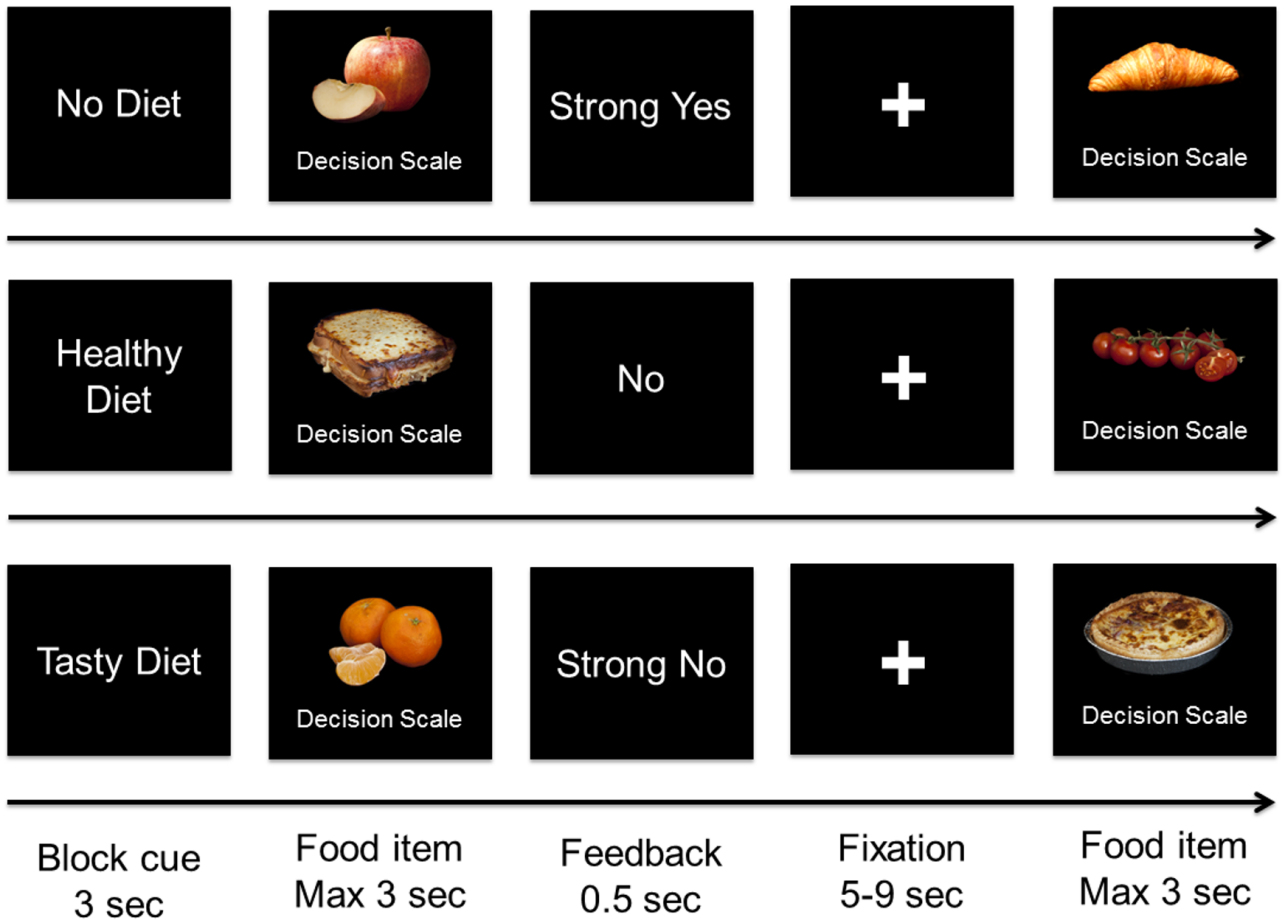
Task summary. Participants made decisions regarding whether or not to eat food items in three attentional conditions: (1) ND condition, (2) HD condition, and (3) TD condition.

Participants evaluated each food item in three attention conditions (healthy diet, tasty diet, and no diet), being informed of which aspect of the food item they were to pay attention to. In the healthy diet condition (HD), the participants were instructed to consider the benefits of eating healthy food. In the tasty diet condition (TD), they were instructed to consider the pleasure of eating healthily, and in the no diet condition (ND, or control condition), they were asked to consider any features of the food that came to their mind. Critically, the instructions emphasized that they should express their own preference, regardless the attention instructions provided at the beginning of the condition. The attention condition was kept the same during for 8 trials at a time, and the beginning of a new condition was announced by means of information displayed on a screen for 3 s (see Fig 1). Participants completed 384 trials in a functional MRI scanner (see technical details below) to assess the activity of their brain while performing the tasks. Visual stimuli were displayed on a screen outside the scanner that participants could see thanks to a mirror system while having the functional MRI data acquired. Each food was displayed once in each experimental condition and the order of presentation of the food items and blocks was fully randomized.

### Measures

Body mass index (BMI) [weight (kg)/height^2^ (m^2^)] was used to identify weight-related health conditions. A BMI ranging from 18.5 to 25 indicates optimal weight, a BMI lower than 18.5 suggests that an individual is underweight, while a BMI higher than 25 indicates that the person is overweight. An individual with a BMI of 30 or more is considered to be suffering from obesity.

### MRI Data Acquisition

Functional neuroimaging was conducted using a 3-Tesla Bruker Medspec 30/80 MRI scanner. To optimize functional sensitivity in the ventromedial prefrontal cortex (vmPFC), a key region of interest, a tilted acquisition in an oblique orientation of 30° to the anterior commissure—posterior commissure plane was used, covering cortical and subcortical areas—except the cerebellum [23]. A T2*-weighted echo planar sequence was used with 30 interleaved 3 mm-thick/1 mm-gap slices (repetition time = 2000 ms, echo time = 30 ms, flip angle = 78,4°, field of view = 192 mm, 64×64 matrix of 3×3×4 mm voxels). A total of 820 functional volumes were collected over four sessions during the course of the experiment. The first six scans in each run were discarded in order to ensure that the longitudinal relaxation time equilibration was achieved. Whole brain anatomical MRI data was acquired using high-resolution structural T1-weighted image (1×1×1mm). A fieldmap acquisition (3D FLASH sequence inter-echo time 4.552ms) was collected in order to estimate and correct the B0 inhomogeneity.

### MRI Data Preprocessing

Image analysis was performed using SPM8 (Wellcome Department of Imaging Neuroscience, Institute of Neurology, London, UK). First, functional EPI images were corrected for slice acquisition time within each volume. Second, motion and distortion were corrected during the realign and unwarp procedure using the fieldmap toolbox. Third, the high-resolution structural T1-weighted image was co-registered to the mean EPI image. Fourth, for all structural MRI volumes, grey matter and white matter images were generated with the new segment option. Fifth, a DARTEL template was generated and spatially normalized into MNI space. This template was used to normalize functional data of each participant. Finally, functional data was spatially smoothed using a Gaussian kernel with a full width at half-maximum of 8 mm.

### MRI Data Analysis

We estimated a general linear model (GLM) of blood oxygen level dependent (BOLD) responses to analyze the data. This model was designed to identify those brain regions in which BOLD activity increases during healthy food choice, as a function of a participant’s BMI. We identified the brain areas in which the BOLD responses increased during healthy food choices as compared to healthy food rejection in the ND condition. The latter condition provided a control condition without drawing attention to healthy food.

Second, we measured the modification of brain activity when attention was drawn to the healthiness of healthy food by examining BOLD responses during healthy food choice in the HD condition as compared to the ND condition.

Third, we measured the modification of brain activity when attention was drawn to the tastiness of healthy food by examining BOLD response during healthy food choice in the TD condition as compared to the ND condition. The modification of BOLD responses as a function of a participant’s BMI was assessed by including the BMI in the model as a covariate.

fMRI data was analyzed using the general linear model (GLM). Our design included 2 categories of food (healthy, unhealthy) x 2 categories of answers (Yes and Strong Yes vs. No and Strong No) x 3 attention conditions (HD, TD, and ND). Motion parameters and session constants were included as regressors of no interest. The GLM was estimated in three steps. First, we estimated the model separately for each individual. Second, we calculated the first-level single-subject contrasts. Third, second-level group contrasts were computed using one-sample t tests on the single-subject contrasts. The BMI was also entered as a covariate. The statistical significance threshold of *p* < .005 (uncorrected for multiple comparisons) was used and we selected a minimum cluster size of 10 voxels across the entire brain, which produces a desirable balance between type I and II error rates [33]. *p*-values significant at *p* < .05 whole brain corrected at the cluster level are also reported.

## Results

### Behavioral results

**Fig 2:**
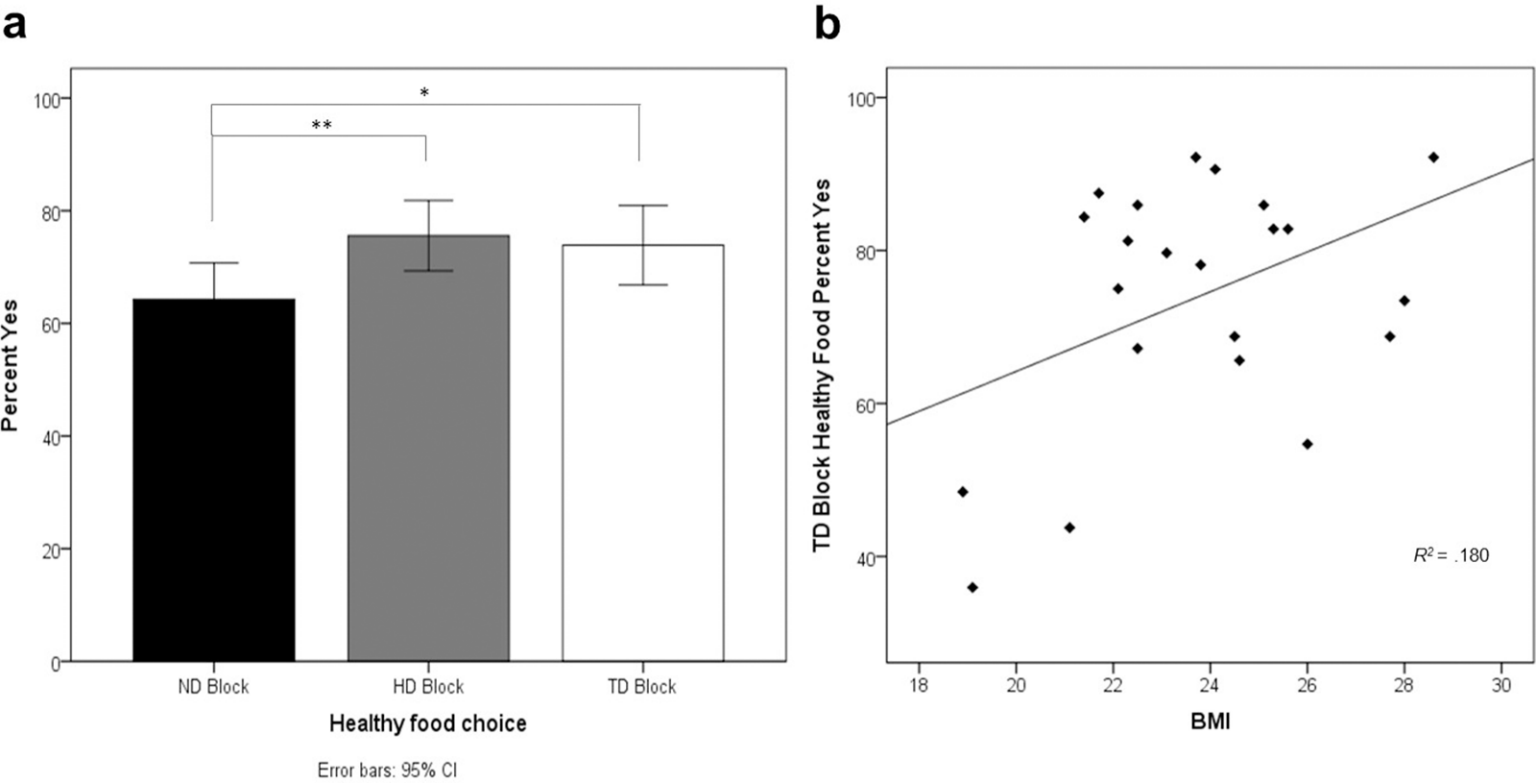
Behavioral results summary. (A) The bar graph shows the percentage of exposures to healthy food when participants responded “yes” or “strong yes” as a function of block type. Asterisks indicate that healthy food choices in the HD and TD blocks differed significantly from ND at *p* < .001 (**) and *p* < .05 (*). Error bars represent 95% confidence intervals. (B) The scatter plots (with best-fitting regression line) show associations between the percentage of healthy food choices in the TD condition and BMI. Each data point represents the percentage of the time that a participant responded “yes” or “strong yes”.

The percentage of the time that participants responded “yes” or “strong yes” to eat healthy food was significantly higher in the HD condition (*t* (21) = 4.28, *p* < .001), and the TD condition (*t* (21) = 2.84, *p* = .01) than in the ND condition. The percentage of healthy food choices was not significantly different between the HD and TD conditions (*p* > .250, two-tailed; See Fig 2A). Considering the effect of demographic characteristics, we did not observe gender-related correlation on the percent of healthy food choices, in the ND (*p* > .250), the HD (*p* = .122) and the TD condition (*p* > .250). However, a significant positive correlation was found between BMI and the percentage of healthy food choices in the TD condition (*R*^2^ = .180, *p* < .05), which was not found in the HD and ND conditions (*p* > .250, two-tailed; see Fig 2B, S1 Data, S1 Table for further details).

### Brain-imaging results

#### Brain regions activated by healthy food choices (vs. healthy food rejects) in the No Diet condition

**Fig 3:**
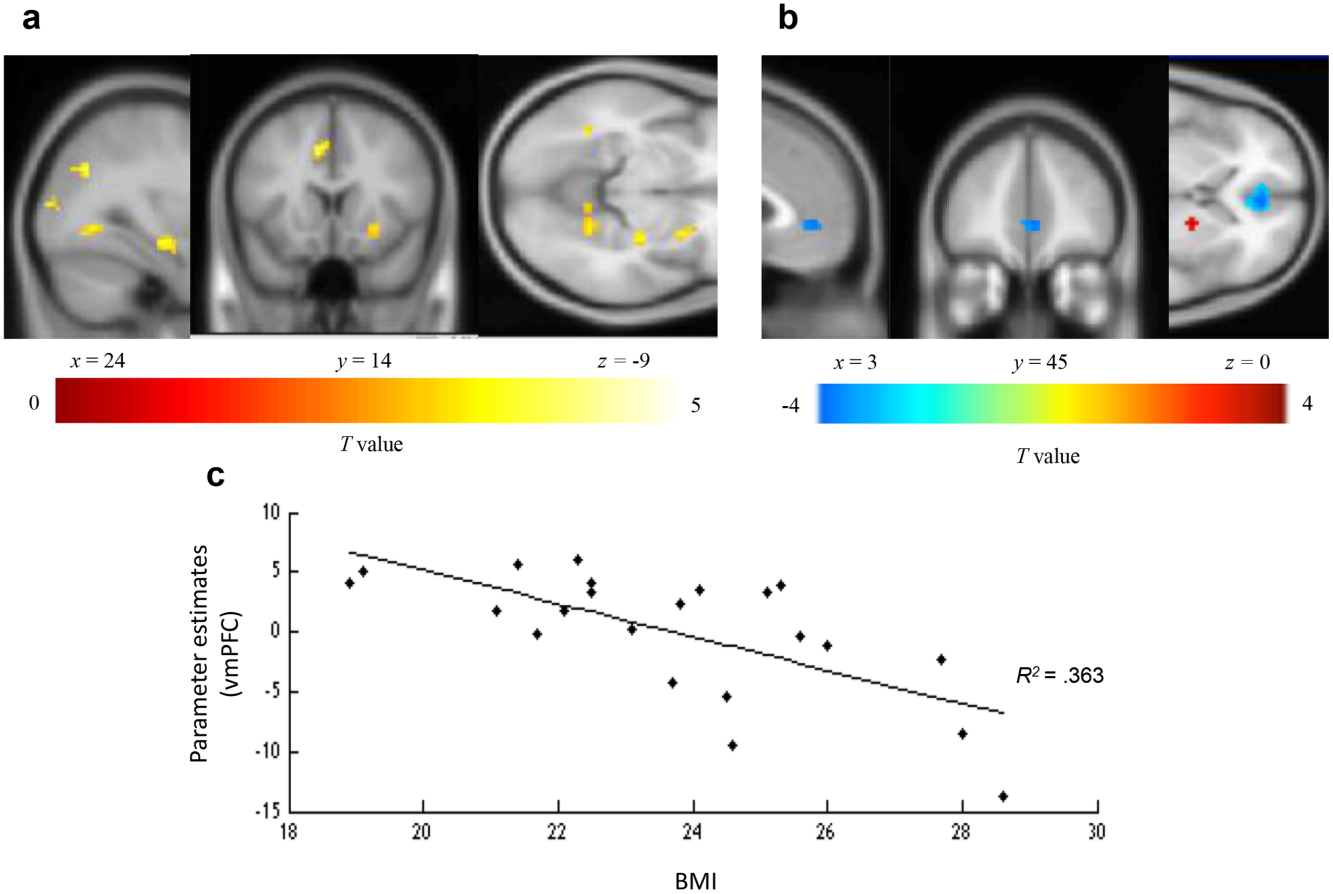
Brain regions showing a BOLD response to healthy food choice compared to healthy food reject in the no diet condition. Contrast images were overlaid onto a group mean anatomy image provided by SPM. (A) Regions where BOLD response correlated with healthy food choice versus healthy food reject independently of BMI. (B) Regions where BOLD response correlated positively (lentiform nucleus, red) and negatively (vmPFC, blue) with BMI during healthy food choice. (C) Graph of parameter estimates (*R*^2^ = .363, p = .003) in the vmPFC peak (x = 3, y = 45, z = 0, *t* = 3.92, *p* uncorrected <. 005) as a function of BMI.

The first step in the functional neuroimaging analysis was to identify those brain regions that were activated by healthy food choices, in the ND condition. We identified the areas in which the BOLD responses were significantly higher during healthy food choices as compared to healthy food reject in the ND condition. We found significantly higher activation in the frontal lobe (left frontal superior medial cortex, right IFG), visual cortical areas (cuneus, right precuneus, left fusiform gyrus), the right hippocampus and the right putamen (see Fig 3A). We also identified a significant positive correlation between BMI and activation in the lentiform nucleus and the precuneus as well as a negative correlation in the right middle temporal gyrus and the vmPFC (anterior cingulate/medial frontal gyrus, Fig 3B and 3C) (see Table 1 and S1 Data for the complete list of activated areas).

**Table 1:** Brain regions more activated in healthy food choice than healthy food reject in the no diet condition as a function of a participant’s BMI.

		MNI coordinates (peak location)				
Region	Lat	x	y	z	T	Cluster size (in voxels)
**Independent of BMI**						
Cuneus	—	0	-75	3	5.21*	503
Precuneus	R	33	-66	33	4.23	14
Hippocampus	R	30	-18	-12	4.11	28
Frontal superior medial gyrus (BA 32)	L	-6	18	42	3.84	15
Inferior frontal gyrus	R	45	24	9	3.56	11
Fusiform gyrus	L	-36	-51	-9	3.56	18
Putamen	R	27	9	-9	3.47	17
**Positively correlated with BMI**						
Precuneus	L	-15	-69	48	4.68	29
Lentiform nucleus	R	18	0	0	3.62	11
**Negatively correlated with BMI**						
Middle temporal gyrus	R	54	-3	-15	4.39	20
Anterior cingulate/medial frontal gyrus	—	3	45	0	4.15*	70

Note: Results for brain regions with a minimum cluster size of 10 voxels, *p* < .005, uncorrected. MNI = Montreal Neurological Institute.

* *p*-values significant at *p* < .05, whole brain corrected at the cluster levels.

#### Brain regions activated in healthy food choice in the Healthy Diet condition (vs. No Diet condition)

**Fig 4:**
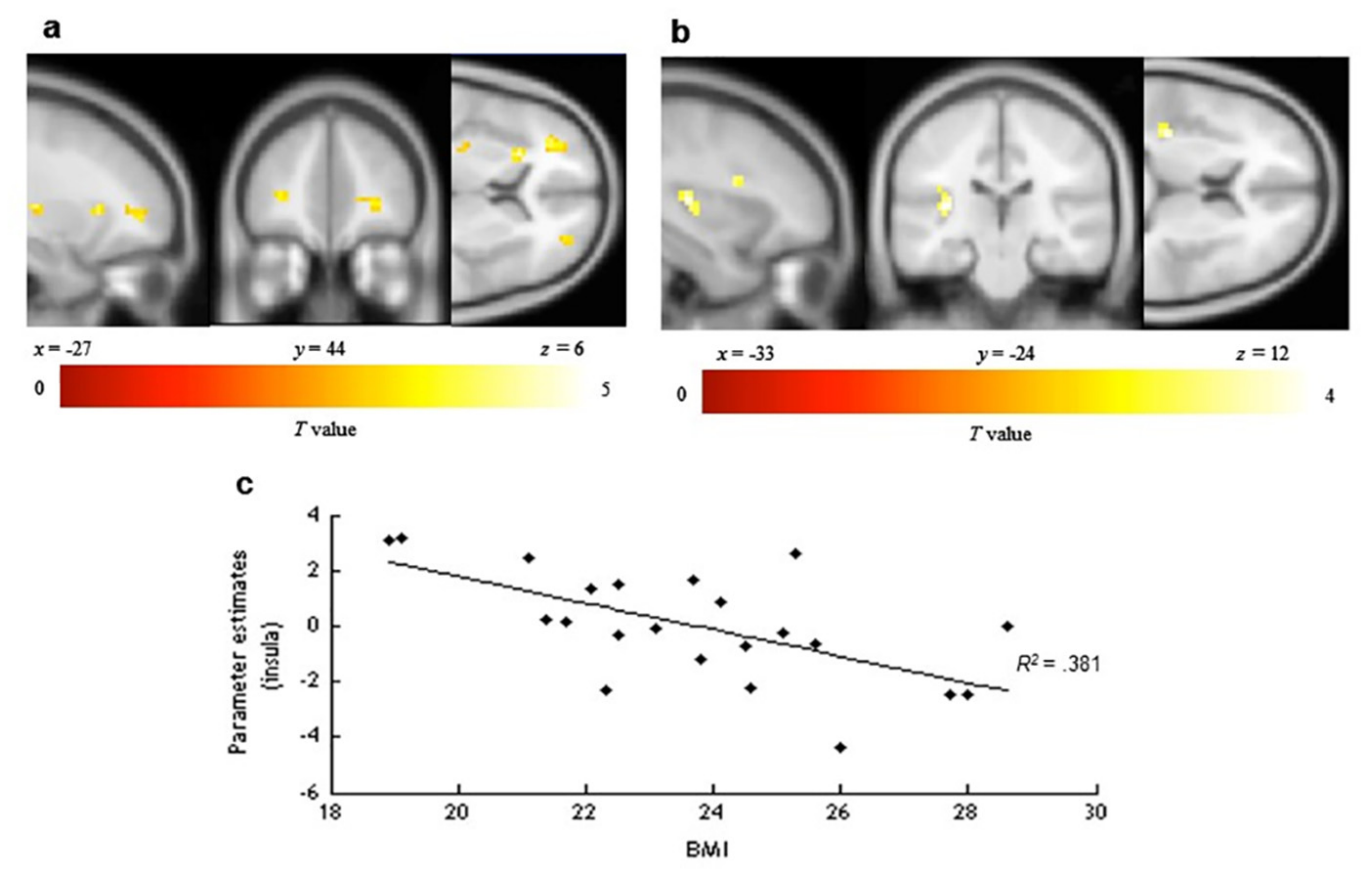
Brain regions with a BOLD response to healthy food choice in the healthy diet condition as compared to the no diet condition. Contrast images were overlaid onto a group mean anatomy image provided by SPM. (A) Regions where BOLD response increased during healthy food choice in the HD condition compared to the ND condition independent of BMI. (B) Regions where BOLD response correlated negatively (left insula, left inferior frontal operculum) with BMI during healthy food choice. (C) Graph of parameter estimates (*R*^2^ = .381, *p* = .002) in the left insula peak (x = -33, y = -24, z = 12, *t* = 4.13, *p* uncorrected <. 005) as a function of BMI.

Next, we identified brain areas that exhibited significantly higher activity during healthy food choices in the HD as compared to during the ND one. Using one-sample t tests at the group level, greater activity was obtained in the OFC (middle frontal gyrus orbital part), the left IFG (BA9), the left insula and the left putamen (Fig 4A, Table 2). A significant negative correlation between BMI and cortical responses was documented in the left insula and the left inferior frontal operculum (Fig 4B and 4C, Table 2, S1 Data).

**Table 2:** Brain regions that were more activated in healthy food choice in the healthy diet condition than the no diet condition as a function of a participant’s BMI.

		MNI coordinates (peak location)				
Region	Lat	x	y	z	T	Cluster size (in voxels)
**Independent of BMI**						
Insula	L	-24	21	6	5.23	15
Putamen	L	-27	-18	9	3.99	11
Middle frontal gyrus, orbital part	L	-30	42	6	3.97	26
Inferior frontal gyrus (BA 9)	L	-51	3	27	3.80	10
Middle frontal gyrus, orbital part	R	30	48	0	3.71	28
**Positively correlated with BMI**						
No regions						
**Negatively correlated with BMI**						
Insula (BA 13)	L	-33	-24	12	4.13	31
Inferior frontal operculum	L	-30	9	24	3.74	10

Note: Results for brain regions with a minimum cluster size of 10 voxels, *p* < .005, uncorrected. MNI = Montreal Neurological Institute.

#### Brain regions more activated in healthy food choice in the Tasty Diet condition (vs. the No Diet condition)

**Fig 5:**
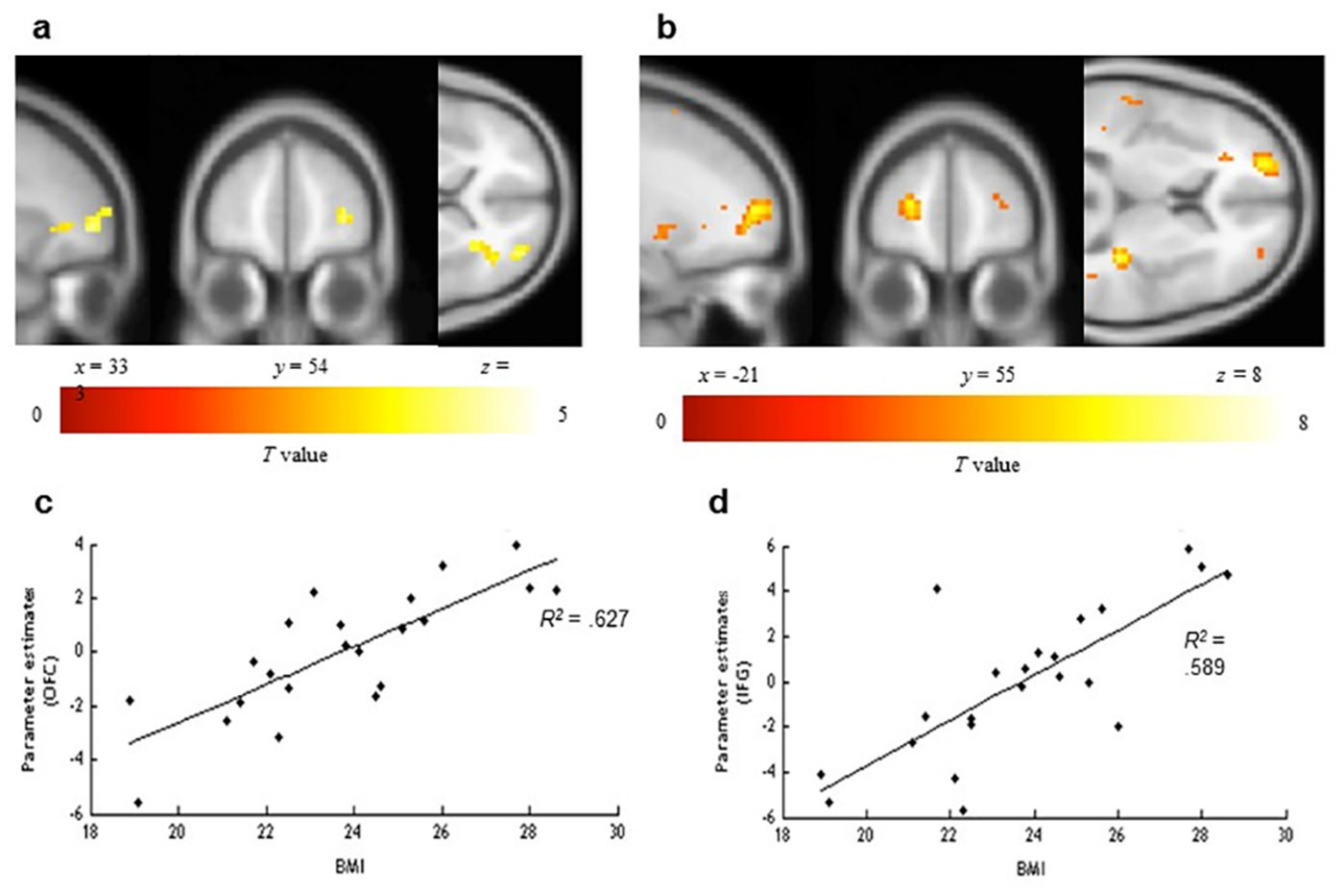
Brain regions showing a BOLD response to healthy food choice in the tasty diet condition compared to the no diet condition. Contrast images were overlaid onto a group mean anatomy image provided by SPM. (A) Regions where BOLD response increased during healthy food choice in the TD as compared to the ND condition independently of BMI. (B) Regions where BOLD response correlated positively with BMI during healthy food choice. (C) Graph of parameter estimates from greater activation (*R*^*2*^ = .627, *p* < .001) in the left OFC peak (x = -21, y = 57, z = 9, *t* = 5.54, *p* uncorrected < .005) as a function of BMI. d. The graph of parameter estimates (R2 = .589, p < .001) in the right IFG peak (x = 36, y = -33, z = 15, *t* = 7.87, *p* uncorrected < .005) as a function of BMI.

Finally, we identified those areas that exhibited a BOLD contrast with healthy food choice in the TD condition as compared to the ND condition. Using one-sample t tests at the group level, greater activity was identified in the right OFC (middle frontal gyrus orbital part), the IFG, the right supplementary motor area, the right inferior parietal lobule and the left caudate nucleus (Fig 5A, Table 3). A significant positive correlation between BMI and cortical responses was observed in the insula, the left OFC (superior frontal gyrus orbital part), the anterior cingulate cortex (ACC), the right IFG, the right inferior parietal cortex, the middle frontal gyrus, the left superior temporal gyrus, the left lentiform nucleus, the precuneus, the left superior frontal gyrus, the right angular gyrus (BA 39), the right middle temporal gyrus, and the right precentral gyrus (BA 4) (Fig 5B and 5C, Table 3, S1 Data).

**Table 3:** Brain regions more activated in healthy food choice in the tasty diet condition than in the no diet one as a function of a participant’s BMI.

		MNI coordinates (peak location)				
Region	Lat	x	y	z	T	Cluster size (in voxels)
**Independent of BMI**						
Inferior frontal gyrus	L	-48	3	24	4.81	17
Supplementary motor area	R	12	-3	63	4.40	14
Inferior frontal gyrus	R	27	30	0	4.09	35
Middle frontal gyrus, orbital part	R	33	54	3	3.96	33
Inferior parietal lobule	R	39	-48	36	3.87	18
Caudate Nucleus	L	-27	-42	12	3.72	10
**Positively correlated with BMI**						
Insula	R	36	-33	15	7.87*	133
Superior frontal gyrus, orbital part	L	-21	57	9	5.54*	94
Anterior cingulate cortex	R	24	30	27	5.25*	68
Inferior frontal gyrus	R	36	12	-12	5.10*	38
Inferior parietal lobule	R	60	-39	27	4.83*	49
Middle frontal gyrus	L	-27	24	33	4.33	17
Superior temporal gyrus	L	-42	-33	15	4.29	21
Lentiform nucleus	L	-27	-6	-9	4.23	28
Precuneus	L	-9	-57	66	4.21*	50
Superior frontal gyrus	L	-18	6	69	4.10	14
Angular gyrus (BA 39)	R	39	-57	21	3.95	10
Insula (BA 13)	L	-36	21	3	3.80*	51
Middle temporal gyrus	R	45	-48	6	3.69	11
Precentral gyrus (BA 4)	R	36	-27	57	3.69	15
Middle frontal gyrus	R	30	42	15	3.48	22
Precuneus	—	0	-60	30	3.46	43
Anterior cingulate cortex (BA 24)	L	-9	27	24	3.14	14
**Negatively correlated with BMI**						
No regions						

Note: Results for brain regions with a minimum cluster size of 10 voxels, *p* < .005, uncorrected. MNI = Montreal Neurological Institute.

* *p*-values significant at *p* < .05 whole brain corrected at the cluster levels.

## Discussion

We hypothesized that: (1) Drawing attention to healthy food would lead to significantly increased activity in those brain areas that are associated with self-control and reward value during healthy food choices, regardless of whether attention is paid to the healthiness or tastiness of food, and help people to make healthy food choices; (2) For higher BMI individuals, focusing on the healthiness decreases the brain activity in these brain areas; (3) Whereas focusing on the tastiness increases their brain activity in these brain areas.

The behavioral and neuroimaging results reported here confirm these hypotheses. Behaviorally, we found that individuals make significantly healthier food choices when their attention is directed toward healthy food (HD and TD conditions). No significant differences in healthy food choice are found between the HD and TD conditions. However, when BMI is considered, a positive correlation between BMI and healthy food choices in the TD condition is observed.

Consistent with the results of previous studies, activity in the OFC/vmPFC correlates with healthy food choice in all conditions [2, 18, 25, 27, 30]. Furthermore, consistent with the view that OFC/vmPFC signals integrate both taste and health value [2, 16], its activity increases during healthy food choice in the HD and TD conditions as compared to the ND one. The presence of both healthy food attention cues is also associated with increased activity in the IFG. The IFG is known to play a central role in inhibition especially during food choice [2, 14, 18–19, 21, 34–35]. The fact that IFG activity also increases when attention is focused on tastiness (TD condition) suggests that this focus does not lead to a more impulsive decision but may help self-control by the valuation of healthy food.

Choosing (vs. not choosing) healthy food based on visual images involves the cuneus (visual cortex), the activation of which is positively correlated with both value and saliency signals during decision-making [26]. However, when BMI is considered, an inverse relationship between BMI and the activity of the OFC/vmPFC is observed. This suggests that the choice of healthy food may be less valued amongst those individuals with a higher BMI. Similarly, when the focus is on healthiness (HD condition), less activity is observed in two of the areas associated with gustatory inferences (insula, frontal operculum) [30]. Such findings suggest that highlighting healthiness in food communications may help to render healthy food less appetizing for high BMI individuals. This is in line with the work of Raghunathan et al. [6] on the UTI, and suggests that this intuition is more pronounced for those individuals with a high BMI [5]. This is also somewhat similar to Hare et al. [2], who showed that for the so-called non-self-controllers, the OFC/vmPFC encodes only the estimated tastiness of food but not its healthiness.

Conversely, when the focus is on the tastiness of healthy food (TD condition), BMI is correlated with an increased activity in reward value areas (OFC/vmPFC, ACC), self-control (IFG), and gustatory inferences (insula) [18, 24, 31]. These results therefore support previous work suggesting that obese individuals are more sensitive to the expected pleasure of consumption as compared to non-obese individuals [15–16, 28, 36–38]. Interestingly, activity in the IFG suggests that the valuation of the tastiness of healthy food results in less impulsive consumption for individuals with high BMI. Previous research has demonstrated that it takes more time and effort to assess the healthiness of food than to determine its tastiness [2, 13], especially for people with poor self-control. By focusing participants’ attention on the tastiness of healthy food, we may have increased their motivation to control themselves and reduced the time to process information, thus making healthy food choices easier to make [10–12]. These preliminary findings should be studied in depth in future studies.

In contrast to previous studies on healthy food choice, no modulation of brain activity was found in the DLPFC. In these studies, participants were explicitly asked to control their desire during decision-making [15, 19–20], or only considered choices of disliked healthy food and no-choices of liked unhealthy food [2]. These situations could require more self-control. By contrast, we find increased activity in the DLPFC when participants reject unhealthy food as compared to when they choose unhealthy food, suggesting that this reject of unhealthy food may be forced, contrary to healthy food choice (see S2 Table).

Our results suggest that focusing attention on both healthiness and tastiness of healthy food may lead consumers to healthier decisions. However, not all individuals are susceptible to the same valuation of healthy food. The valuation of health benefits may have a counterproductive effect on those individuals with high BMI by reducing the expected pleasure of consumption, as suggested by the negative correlation between BMI and the activity in gustatory and reward value areas in the HD condition. However, those individuals with a high BMI do not necessarily have less capacity for self-control than those with a low BMI. They may even be in a position to control themselves more effectively whenever the pleasure of eating healthy food is promoted, as suggested by the positive correlation found between BMI and the activity in self-control related brain areas in the TD condition. Future research is needed to test these relationships directly.

Despite our encouraging results, we would like to highlight some limitations of our study that should to be taken into consideration. First, we did not consider gender, socioeconomic, and other environmental factors in our analysis (e.g. price, social situation, income, education, location, etc.) that are known to influence food perception and behavior [39–42]. To guide interventions and policy changes, future research will need to include these variables and examine the effect of focusing on the tastiness of healthy food in more “ecological” environments. Moreover, this study was conducted on ‘optimal’ weight and overweight participants (as defined by their BMIs). Replicating our study by comparing obese individuals to non-obese ones is therefore warranted.

Second, in our study we did not use a constraining task: that is, the participants were not explicitly asked to control themselves. For this reason, we can only go so far as to conclude that brain networks associated with self-control are more active in people with a higher BMI in the TD condition, thus suggesting that their self-control may be facilitated by external attention cues on the tastiness of healthy food. The results of this study lead to the hypothesis that self-control can be improved by focusing on the tastiness of healthy food, which need further research.

Third, we did not measure whether promoting the pleasure of eating food affects the unhealthy = tasty intuition (UTI). It would be interesting to test this relationship.

Fourth, in our study, we explicitly asked participants to focus their attention on the tastiness or on the healthiness of food during their choices. Future studies might also compare the effects of promoting each of these two attributes of healthy food directly in advertising campaigns and packaging on food behaviours, and the correlations with the BMI of participants.

Despite these limitations, we are confident that our study provides a solid base for further research on the role of pleasure in healthy food choices and on self-regulation in general and helps promote the use of consumer neuroscience in order to better understand food choices.

## Supporting Information

S1 Data Behavioral datasets and fMRI group analysis. (ZIP)

S1 Table Correlation between BMI and the percentage of healthy food choice. (PDF)

S2 Table Brain regions more active in unhealthy food reject than unhealthy food choice as a function of a participant’s BMI. (PDF)
