# Supplementary material for: Health and Pleasure in Consumers' Dietary Food Choices: Individual Differences in the Brain's Value System
Source: PLoS One. 2016 Jul 18;11(7):e0156333. doi: 10.1371/journal.pone.0156333 (PMC4948867; doi:10.1371/journal.pone.0156333)
Supplement: S1 Table — (PDF) [file pone.0156333.s002.pdf]

Pearson Correlation [a.]

	BMI	ND Block HF	HD Block HF	TD Block HF	ND Block UF	HD Block UF	TD Block UF
**BMI**	1	.106	.152	.424*	.488*	-.304	-.239
		.639	.500	.049	.021	.168	.285
**ND Block HF**	.106	1	.626**	.462*	-.205	-.319	-.135
	.639		.002	.031	.359	.147	.550
**HD Block HF**	.152	.626**	1	.814**	.104	-.461*	-.348
	.500	.002		.000	.645	.031	.113
**TD Block HF**	.424*	.462*	.814**	1	.350	-.325	-.120
	.049	.031	.000		.110	.139	.593
**ND Block UF**	.488*	-.205	.104	.350	1	-.077	-.021
	.021	.359	.645	.110		.735	.927
**HD Block UF**	-.304	-.319	-.461*	-.325	-.077	1	.709**
	.168	.147	.031	.139	.735		.000
**TD Block UF**	-.239	-.135	-.348	-.120	-.021	.709**	1
	.285	.550	.113	.593	.927	.000	

HF = Healthy Food, UF = Unhealthy Food *. Correlation is significant at the .05 level (2-tailed).

**. Correlation is significant at the .001 level (2-tailed). [a.] Listwise N = 22
