# Supplementary material for: Health and Pleasure in Consumers' Dietary Food Choices: Individual Differences in the Brain's Value System
Source: PLoS One. 2016 Jul 18;11(7):e0156333. doi: 10.1371/journal.pone.0156333 (PMC4948867; doi:10.1371/journal.pone.0156333)
Supplement: S2 Table — (PDF) [file pone.0156333.s003.pdf]

Region	Lat	MNI coordinates (peak location)			T	Cluster size (in voxels)
		x	y	z		
**Independent of BMI**						
Precuneus (BA 19)	L	-30	-72	45	4.08	14
Dorsolateral prefrontal cortex (BA 9)	R	51	27	33	4.01	13
Angular gyrus	L	-45	-60	30	3.80	26
Orbitofrontal cortex	R	51	45	0	3.25	10
**Positively correlated with BMI**						
No regions significant						
**Negatively correlated with BMI**						
Middle occipital gyrus	R	39	-72	18	4.68*	57
Lentiform nucleus	L	-24	-15	0	4.05	44
Supplementary motor area	—	0	15	57	3.93	25
Precentral gyrus (BA 6)	L	-33	-9	69	3.91	10
Middle frontal gyrus	R	30	6	45	3.90	28
Cuneus (BA 18)	—	-6	-78	15	3.82	40
Hippocampus	L	-30	-36	6	3.71	26
Middle frontal gyrus	R	27	21	30	3.65	46
Inferior parietal lobule	L	-51	-30	45	3.30	16

Note: The table presents results only for those brain regions with a minimum cluster size of 10 voxels, $p < .005$, uncorrected. MNI = Montreal Neurological Institute. * *p*-values significant at $p < .05$ whole brain corrected at the cluster levels.
